# Autism Detection in Children: Integrating Machine Learning and Natural Language Processing in Narrative Analysis

**DOI:** 10.3390/bs14060459

**Published:** 2024-05-29

**Authors:** Charalambos K. Themistocleous, Maria Andreou, Eleni Peristeri

**Affiliations:** 1 Department of Special Needs Education, Faculty of Educational Sciences, University of Oslo, 0313 Oslo, Norway; charalampos.themistokleous@isp.uio.no; 2 Department of Speech and Language Therapy, University of Peloponnese, 24100 Kalamata, Greece; 3 School of English, Aristotle University of Thessaloniki, 54124 Thessaloniki, Greece; eperiste@enl.auth.gr

**Keywords:** autism spectrum disorder, narrative production, expressive vocabulary, machine learning, natural language processing, early diagnosis

## Abstract

Despite the consensus that early identification leads to better outcomes for individuals with autism spectrum disorder (ASD), recent research reveals that the average age of diagnosis in the Greek population is approximately six years. However, this age of diagnosis is delayed by an additional two years for families from lower-income or minority backgrounds. These disparities result in adverse impacts on intervention outcomes, which are further burdened by the often time-consuming and labor-intensive language assessments for children with ASD. There is a crucial need for tools that increase access to early assessment and diagnosis that will be rigorous and objective. The current study leverages the capabilities of artificial intelligence to develop a reliable and practical model for distinguishing children with ASD from typically-developing peers based on their narrative and vocabulary skills. We applied natural language processing-based extraction techniques to automatically acquire language features (narrative and vocabulary skills) from storytelling in 68 children with ASD and 52 typically-developing children, and then trained machine learning models on the children’s combined narrative and expressive vocabulary data to generate behavioral targets that effectively differentiate ASD from typically-developing children. According to the findings, the model could distinguish ASD from typically-developing children, achieving an accuracy of 96%. Specifically, out of the models used, hist gradient boosting and XGBoost showed slightly superior performance compared to the decision trees and gradient boosting models, particularly regarding accuracy and F1 score. These results bode well for the deployment of machine learning technology for children with ASD, especially those with limited access to early identification services.

## 1. Introduction

Autism spectrum disorder (ASD) is a neurodevelopmental disorder that impairs social communication and language development in children, hindering children’s social and academic well-being [1,2,3]. ASD prevalence is on the rise, particularly in Europe and the USA [4,5,6]. Hence, early diagnosis is of paramount importance to enable timely therapy interventions that enhance development, language, and communication in children with ASD, underscoring the critical need for diagnosis before the age of three [7,8,9].

Despite the urgent need for early diagnosis, ASD screening often depends on teacher and parent familiarity with symptoms, leading to delayed diagnoses. Consequently, a growing number of children seek consultation in adolescence, which can have detrimental effects on intervention efficacy [10]. Also, ASD assessment is a complex, stressful, and time-consuming process for the child. It depends on clinical expertise in administration and scoring, which can hinder objective diagnostic outcomes. Thus, families with children with ASD lacking access to specialists or knowledge to navigate the clinical and administrative process are especially disadvantaged [11,12,13]. This study aims to provide a quick and easy machine learning (ML) model for the early screening of individuals with ASD, which can potentially be applied for the early screening and identification of children with ASD.

This study investigates the narrative production and expressive vocabulary skills of Greek-speaking children with ASD. In Greece, the median age of ASD diagnosis is approximately seven years, with a further two-year delay for lower-income families residing in remote, rural areas [14]. Around 75% of ASD diagnoses occur after the age of six years, while less than 25% are made during preschool age. Several factors contribute to delayed autism diagnosis in Greece, including limited awareness of ASD symptoms among caregivers and a lack of understanding regarding subtle language impairments, which can be early indicators of autism [15]. Also, Greek caregivers of children with ASD exhibit the lowest average scores in language symptom endorsement compared to caregivers in Italy, Japan, Poland, and the USA [16].

### 1.1. Narrative Performance in ASD

Research in the language development of children with ASD provides testable predictions regarding the language features that may be severely impacted during early child language acquisition [17]. For instance, several studies have demonstrated that a substantial proportion of children with ASD exhibit significant delays in expressive vocabulary skills, primarily attributed to limited verbal imitations and gestures for initiating joint attention [18,19,20,21,22]. Language assessment of children with ASD has historically included tools whose administration is usually time-consuming, thus hindering the early identification of effective intervention goals [23].

The elicitation of coherent discourse through picture-based narrative tasks has been a widespread practice of language assessment in young children, since narratives represent a universal form of discourse not being subject to the cultural biases commonly seen in standardized language tests [19,23,24,25,26]. Furthermore, narratives can be very informative about a wide range of language skills, ranging from the use of lexical and morphosyntactic aspects of language (also known as microstructure), to the quality of the overall story structure, including the use of cohesive ties and affective language, such as internal state terms, also known as constituting the story’s macrostructure [25,27,28]. Children with ASD exhibit more marked weaknesses than typically-developing (TD) peers in both the micro- and macro-level of the story’s organization, and especially in language elements with strong pragmatic import, such as pronouns, affective terms, and figurative language [29,30,31]. Differences between ASD and TD peers in narrative performance have so far been reflected in children with ASD’s lower semantic quality [32], lower production of internal state terms [25,33], and limited number of causal conjunctions (e.g., with, because) used for the instantiation of relations between story events [24]. Existing evidence also suggests that pragmatic limitations in ASD cannot be overcome by good lexical and syntactic language skills [3,26], which gives added value to the use of narration as a language assessment task for children with ASD.

While narrative sampling provides valuable insights into the language characteristics of ASD and should be a standard diagnostic tool, its widespread adoption is hindered. Currently, analyzing narrative production in children with ASD remains time-consuming and labor-intensive. Eliciting the narrative, manually coding, annotating, and analyzing utterances often requires specialized expertise. Furthermore, variability in coding methods and the potential for subjective interpretations have contributed to inconsistent findings regarding the specific nature of language weaknesses in ASD [34]. Moreover, while it is generally understood that basic research on the language characteristics of ASD should translate into applied research to develop tools for early diagnosis, more than two thirds of funding worldwide is still directed towards basic research [35,36]. Despite a surge in research on language in ASD worldwide over the past two decades, and a strong focus on early diagnosis, there has been minimal change in the proportion of applied research dedicated to directly enhancing diagnostic tools for this neurodevelopmental condition.

While quantitative measures derived from individual language paradigms have been linked to ASD specifiers, like the co-occurrence of ASD and syntactic impairment [23,26,37,38,39], there is a critical need for objective, automated assessments of children with ASD’s language skills and rigorous cross-disciplinary methodologies to provide reliable markers of ASD for early assessment and diagnosis. Therefore, the identification of reliable markers of ASD in language and the early detection of ASD require novel technologies and ML paradigms capable of generating behavioral targets that effectively differentiate ASD from non-ASD features in children.

### 1.2. The Current Study

The aim of this study is to tackle the critical need for the accurate and early diagnosis of ASD by developing a reliable and practical ML model applied to ASD children’s narrative and vocabulary data. Unlike traditional methods that can be subjective and time-consuming, requiring lengthy evaluations, an ML approach offers the potential for standardized, rapid, and widely applicable screening [40]. For example, in our previous research, we have demonstrated that combined natural language processing (NLP) and ML pipelines have the potential to identify Swedish patients with mild cognitive impairment and Alzheimer’s disease from healthy controls [41,42] and also subtype patients with primary progressive aphasia into PPA variants (non-fluent PPA, semantic PPA, and logopenic PPA) using an ML model [40]. Also, we had demonstrated that NLP, automated part-of-speech (POS) tagging, and syntactic parsing can enable the identification of agrammatism in patients with the non-fluent variant of PPA [43]. 

Implementing ML approaches to language data in ASD could equip educators, clinicians, and others with a powerful tool to identify children at risk for ASD, ultimately facilitating earlier access to crucial interventions. An ML-based tool is crucial for educators and clinicians because it addresses several challenges they face in the classroom and in clinical settings when working with children with ASD. Educators often encounter diverse learning needs among their students, including variations in communication abilities. Identifying children at risk for ASD, who may require additional support, can be challenging due to the broad spectrum of symptoms [13,44] and the overlap with other developmental conditions [45].

Additionally, educators may struggle to access timely assessments and interventions, leading to delays in addressing ASD students’ needs effectively. By providing educators and clinicians with an artificial intelligence (AI)-based screening tool that analyzes language profiles for early identification of ASD, we will be able to streamline the referral process, ensuring that children receive appropriate interventions promptly. This will not only support the individualized learning needs of children with ASD but will also promote a more inclusive and supportive learning environment for all children with ASD attending mainstream schools. Finally, by focusing on narrative and expressive vocabulary skills, the proposed model targets key areas of communication often affected in ASD [26,32,46,47]. In the current study, we offer a novel comprehensive ML framework to identify narrative production and expressive vocabulary as domains that distinguish children with ASD from age-matched TD children.

Research in markers of ASD cross-linguistically indicates a pertinent need for an objective and easy-to-apply method for discerning children with ASD from TD children. Modern AI technologies can offer both automatic language biomarkers of ASD, namely language features that characterize individuals with ASD, and reliable classification models towards this direction. Despite the acknowledgment of AI’s potential in leveraging biomarkers for a data-driven approach to ASD classification [48], the predominant reliance on behavioral observation data remains a challenge. Unlike genetics and neuroimaging scans, which follow established protocols for collection and analysis, behavioral observational data encounter difficulties in capturing the dynamic changes in an individual’s behavior [49]. While AI is increasingly being employed to autonomously interpret information from the environment, the combination of AI and behavioral data holds promise in overcoming the challenges associated with data collection during the screening and diagnostic phases. Notably, despite individual studies focusing on specific AI methods in ASD [50,51], a study of AI technology that would distinguish children with ASD from TD peers by placing emphasis on behavioral aspects of children’s performance, such as narrative production and vocabulary, is currently lacking. This study aims to leverage the capabilities of AI to develop a reliable and practical model for distinguishing children with ASD from TD peers based on their narrative and expressive vocabulary skills. This approach could potentially offer a valuable tool for early and accurate diagnosis, facilitating timely interventions and support for children with ASD.

## 2. Materials and Methods

### 2.1. Participants

The study included in total 68 children with ASD (53 males) and 52 TD children (41 males). The children with ASD were recruited from the geographical region of Macedonia in northern Greece, and were referred by Centers for Differential Diagnosis, Assessment, Counseling, and Evaluation (KEDASY) that constitute the official state centers responsible for the diagnosis and assessment of autism and other developmental disorders in Greece. All children received a formal clinical diagnosis of autism at preschool age at a KEDASY on the basis of the DSM-V and ICD-10 criteria [52,53], as well as a record review conducted by teams with diverse expertise (psychiatrist, clinical psychologist, specialized educator, social worker, speech language pathologist). Finally, the Autism Diagnostic Interview—Revised [54] was administered to children diagnosed with ASD. Children with coexisting intellectual and language difficulties were excluded from the sample. The children with ASD attended mainstream classes in public schools. TD children were selected for normal hearing; no speech, emotional, or behavioral problems; and no observed neurological, articulation, and phonological deficits. They were all recruited from mainstream public schools in northern Greece. Experimental data were collected following children’s parents’ formal written consent. Children’s Full-Scale IQ (FSIQ) scores were estimated using the Greek version of the Wechsler Intelligence Scale for Children, 5th Edition [55], Greek version by [56]. Table 1 below presents the mean age and the FSIQ scores of each group. The mean ages and standard deviations, as well as the age ranges in Table 1, represent the children’s years and months. The two groups did not differ in either age, χ^2^ = 5.477, *p* = 0.571, or FSIQ, χ^2^ = 9.369, *p* = 0.195. All study procedures were conducted in accordance with the Declaration of Helsinki and approved by the institutional review board (IRB) and ethics committee of the Aristotle University of Thessaloniki (IRB protocol number: 39928). 

### 2.2. Procedure

Narrative production. We analyzed the narrative (telling) productions of each child in the four pictured stories of the Multilingual Assessment Instrument for Narratives [57], Greek version by [58]; retrievable from https://main.leibniz-zas.de/ (accessed on 3 September 2014). The four stories were comparable in terms of both stimuli (i.e., picture-based sequences) and story structure complexity. Children were seated individually in a quiet room in front of a computer workstation and opposite the examiner (third author). The parents of the children were not allowed in the room to eliminate as many distractions as possible. The children were informed by the examiner that she has never seen the stories before, and that they should carefully view the pictures in order to be able to tell a story in their own words. The six-picture sequence was visible to the children while telling the story and was only removed after the child finished speaking. The children received no prompts from the examiner and there was no time limit set. Immediately following the telling of the story, the children were given specific feedback on their participation (e.g., “You did a great job of telling the story”; “Thanks for being such a good storyteller”). Children’s storytellings were digitally recorded on an Olympus DS-30 Digital Stereo Voice Recorder. The narratives were then manually transcribed at the word level by the third author. All transcripts were then checked by the second author, reaching high inter-rater reliability (r = 0.95). The transcripts were next fed into the AI system.

Expressive vocabulary. The children’s expressive vocabulary in Greek was assessed through an expressive vocabulary test, which has been standardized for 3- to 10-year-old Greek-speaking monolingual children [59]; adaptation from [60]. It includes 50 black-and-white pictures of common objects that each child was asked to name individually. Each correct answer earns one point, with a maximum score of 50. The test was terminated when the participant failed to respond correctly to five consecutive trials. The administration of the task lasted approximately 10 min for each child.

### 2.3. Machine Learning 

We developed a robust computational modeling approach by integrating several pipelines for the automatic analysis of the narrative data and the development of the ML models for the classification. First, we manually transcribed the four speech recordings from both groups (ASD and TD) by the second and third author. Next, we utilized NLP techniques to extract relevant linguistic features from the transcripts. Finally, we developed and implemented various ML models to identify patterns within the linguistic data. This section details the steps involved in this process.

### 2.4. Feature Engineering

We combined measures from NLP analysis and embedding measures from a Bidirectional Encoder Representations from Transformers (BERT) language model [61]. These would serve as numerical inputs to build classification or prediction models. Researchers might focus on specific subgroups to identify those most helpful for a task (like distinguishing children with ASD from TD peers).

1. NLP analysis. We employed Open Brain AI [62] to elicit measures from the text transcripts. These measures include the following information. We evaluated both count measures and word ratio measures that represent the proportion of a specific feature relative to overall text length (word count). This helps control for differences in the length of text samples. The analysis included the following language features in children’s narratives:
  i. Grammatical Features. We included information about the word classes (parts of speech), e.g., counts of adjectives, adverbs, nouns, pronouns, verbs, proper nouns, determiners, and numerals. We also included two types of engineered measures, namely, content and function words. Content words constitute a group measure of words with significant meaning (nouns, verbs, adjectives, some adverbs) while function words constitute a group measure of words with primarily grammatical roles (conjunctions, articles, pronouns);
  ii. Syntactic and Dependency Relations. We included information such as counts of adjectival modifiers, nominal subjects, direct objects, clausal complements, and prepositional modifiers, as well as conjunctions, such as counts of coordinating conjunctions and subordinating conjunctions;
  iii. Focusing on Grammatical Elements. We included count measures of auxiliaries, particles, and case markers;
  iv. Semantic Features. We included count measures of named entities, such as semantic references to persons and locations. We also included count measures of other semantic categories, such as counts of events, time references, date references, and quantities;
  v. Text Complexity and Style. We included information on word counts, including measures of character and syllable usage. Also, we provided information on vocabulary richness and diversity, such as the type–token ratio (TTR), corrected TTR, the summer index, word density, Maas’s TTR, Mean Segmental_TRR, and Herdan’s c [62].
2. BERT embeddings. We extracted BERT embeddings from textual data using the “nlpaueb/bert-base-greek-uncased-v1” model [63]. This deep learning-based feature extraction aimed to capture complex patterns and semantic information from the text, which often need to be discernible through traditional NLP analysis. The resulting embeddings were combined with other numerical features from the dataset, creating a comprehensive feature set.

### 2.5. Addressing Data Scarcity and Imbalance

Recognizing the limitations posed by the small size of our dataset, we employed random over-sampling (ROS), a powerful novel computational approach to balance the classes [64,65]. This approach helped mitigate the model’s bias towards the majority class, a common issue in imbalanced datasets. Additionally, the group five-fold cross-validation was utilized with “child id” as the grouping factor to avoid data leakage and provide a more reliable assessment of model performance. A median-based imputation strategy was adopted to tackle the issue of missing values. This step ensured that our models received a complete dataset, which is crucial for accurate prediction. Furthermore, we standardized the non-BERT features to ensure uniformity in scale.

### 2.6. Model Comparison and Selection

As each ML model captures different types of information on a certain dataset, it is critical to determine the one that provides the best outcomes through a rigorous model comparison approach. We included gradient boosting, decision trees, hist gradient boosting, and XGBoost.

1. Gradient boosting is an ensemble learning method that combines multiple weak learners to make predictions. It is a sequential algorithm, which means that it builds one weak learner at a time, using the information from the previous weak learners to improve the next weak learner. Gradient boosting is a viable choice for problems with a lot of data, and the features are high-dimensional [66,67];
2. Decision trees are tree-like structures representing a series of decisions and their possible consequences. They are used for classification and regression tasks. Decision trees are a viable choice for problems where the data is easily interpretable and a few key features are essential for making predictions [66,67,68];
3. Hist gradient boosting is a variant of gradient boosting that uses histograms to represent the features. This makes it more efficient than gradient boosting, especially for problems with a lot of data and high-dimensional features [69];
4. XGBoost is a widespread implementation of gradient boosting known for its speed and accuracy. It uses several techniques to improve the performance of gradient boosting, such as using a more efficient tree-splitting algorithm and regularization to prevent overfitting [70].

Table 2 summarizes the characteristics of each of these ML models.

Achieving an optimal model performance hinges on selecting the most effective configuration of hyperparameters for each one of these models. A grid search with cross-validation was implemented to evaluate and compare the performance of the different ML models by finding the optimal hyperparameters for each model using grid search and calculating the evaluation metrics.

The grid search offers a systematic approach to evaluate the hyperparameters of the models. Specifically, we employed GridSearchCV, a scikit-learn function that automates an exhaustive hyperparameter search [66,67]. First, we specified the hyperparameters of the models with possible values. This creates a grid of all possible combinations. GridSearchCV iterates through each combination in the grid. Each combination splits data into training and validation sets and trains a model instance with the current hyperparameter combination after evaluating the model’s performance on the validation set using accuracy measures. After assessing all combinations, GridSearchCV identifies the hyperparameter combination that yields the best performance on the validation set. This combination is considered the “best model” based on the explored search space.

The hyperparameters are essentially control knobs that influence the learning process of a model. They are distinct from model parameters, which are learned from the training data. Depending on the measures, we evaluated different hyperparameters, such as the learning rate, which controls the step size taken by the model during training, impacting the speed and convergence of learning, and the number of trees (in random forests), which determines the model complexity and potential for overfitting. The grid search employs an exhaustive evaluation strategy to identify the optimal hyperparameter configuration. The grid search offers a systematic way to identify well-performing hyperparameter configurations, particularly when the search space is of manageable dimensionality.

Subsequently, the ML models were evaluated by assessing the following performance metrics, namely, their accuracy, F1 score, precision, recall, ROC AUC, and Cohen’s kappa. These metrics provide a multi-faceted assessment of each model’s predictive power and its ability to handle specific complexities like class imbalance. Additionally, ROC AUCs (area under the receiver operating characteristics curves) were generated, offering a visual representation of each model’s capacity to discriminate between classes at various classification thresholds.

## 3. Results

**Figure 1:**
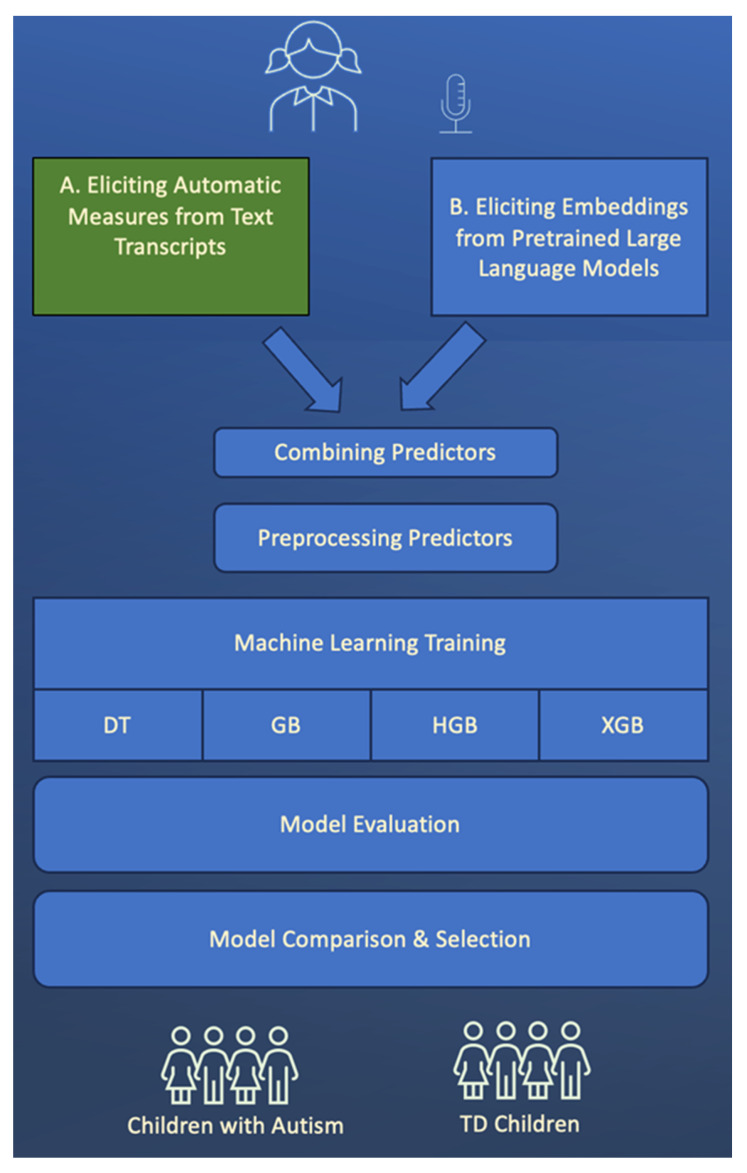
Data engineering, model design, training, and selection processes; the design allows the ML model to predict the diagnostic status (ASD vs. TD) of the child from narrative production and expressive vocabulary. DT: decision trees, GB: gradient boosting; HGB: hist gradient boosting; and XGB: XGBoost.

**Figure 2:**
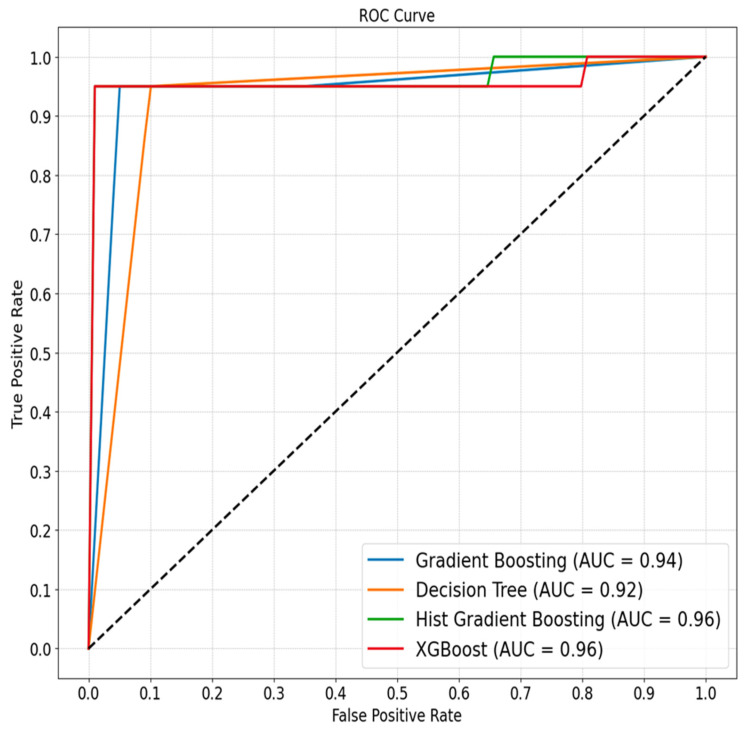
The ROC AUC curve was produced by ML models for distinguishing ASD from TD children.

The ML models were trained on the ASD and TD children. The findings show that narrative production combined with expressive vocabulary distinguishes ASD from TD children, achieving an accuracy of around 96% (see Figure 1, Table 3). This highlights the role of language in informing diagnosis of ASD. All four models performed well on this binary classification task, with hist gradient boosting and XGBoost showing marginally better performance across most metrics. The best model choice might depend on the specific application and the cost of false positives versus false negatives. For instance, a model with higher recall might be preferred in ASD diagnosis (where missing a positive case could be critical). On the other hand, in a spam detection scenario (where false positives could be more disruptive than false negatives), a model with higher precision might be more desirable (Figure 2).

Hist gradient boosting and XGBoost showed slightly superior performance compared to the decision trees and gradient boosting models, particularly regarding accuracy and F1 score. This is due to their advanced algorithms, which effectively analyze complex data patterns. The perfect precision (1.0) for hist gradient boosting and XGBoost indicates that they are extremely likely to be correct when these models predict a positive class. The ROC AUC values were high for all models (see Figure 1), particularly for hist gradient boosting and XGBoost, reinforcing their ability to distinguish between ASD and TD children. The consistent recall across all models suggests they are equally effective at identifying positive cases.

More specifically, the accuracy indicates the proportion of correct predictions (both true positives and true negatives). An accuracy of 0.95 for the decision trees and gradient boosting models and 0.975 for the hist gradient boosting and XGBoost models indicate a high level of overall correctness in distinguishing between ASD and TD children based on the narrative production and expressive vocabulary performances. 

The F1 score is the harmonic mean of precision and recall, balancing these two metrics. A high F1 score, as seen across all models (ranging from 0.95 to approximately 0.974), suggests a good balance between precision and recall, and corroborates the accuracy. 

The ROC AUC measures the ability of a classifier to distinguish between classes and is used as a summary of the ROC curve. Values range from 0 to 1, with a higher score indicating better classification capabilities. The scores here ranged from 0.94375 to 0.96750, indicating good discriminative ability for all models.

Finally, the two last measures reported the precision and the recall, both displaying a value closer to 1, which indicates good precision and recall. Specifically, the precision is the ratio of correctly predicted positive observations to the total predicted positives. High precision (0.95 to 1.0) across all models indicates a low rate of false positives. The recall measures the proportion of actual positives that were correctly identified. The consistent recall of 0.95 for all models suggests they identify the positive class well.

## 4. Discussion

In this study, we employed ML model comparison techniques to identify classifiers with high accuracy that could distinguish ASD from age-matched TD children on the basis of the children’s narrative production and expressive vocabulary performances. The top performing models were the hist gradient boosting and XGBoost algorithms that provided high classification accuracy, namely 96.75% and 96% accuracy, respectively, thus robustly differentiating children with ASD from their TD peers. Moreover, an exceedingly small number of children were misclassified, which further suggests that morphosyntactic, semantic, and pragmatic measures of children’s narrative production, coupled with expressive vocabulary, can achieve high classification accuracy for children with ASD. 

### 4.1. Significance of ML Classsifiers in Early Detection of ASD

This approach demonstrates the feasibility of integrating ML in educational settings for diagnosing ASD in children. Current language assessment procedures in ASD present significant challenges of being time-consuming, requiring extensive training for clinicians to objectively and accurately code, analyze, and evaluate the complex language data. Early educational and behavioral intervention is critical for improving children’s quality of life and enhancing their social and personal development [71,72]. The resulting delays in diagnosis can be detrimental, as early intervention with speech therapy has been shown to have the most significant impact on ASD children’s future development and quality of life [73]. Thus, the present study provides an automated method to screen children for ASD which is fast in application and reliable. The high accuracy of the models suggests that they could be used as a quick and easy assessment tool for children suspected of having ASD. A key advantage is that the training process only needs to be performed once. Once trained, the model can be used to evaluate many new individuals efficiently.

Our AI-based data analytics to identify ASD on the basis of the children’s narrative and vocabulary skills may be viewed as part of a broader AI approach to improving objectivity in the early diagnosis of ASD, as well as to enhancing access to clinical services and educational opportunities for these individuals. AI-based designs have so far mainly capitalized on cognitive and behavioral phenotypes in children with ASD, including stereotypical behaviors [74], diagnostic measures such as the Autism Diagnostic Observation Schedule (ADOS) [75], the Autism Diagnostic Interview—Revised (ADI—R) [54], or the Childhood Autism Rating Scale (CARS) [76,77,78]. Interestingly, ML classifiers have been recently developed to predict which types of teacher communication strategies are more likely to generate positive responses in seven students with ASD in the classroom [79]. Though the collected language-based data fed into the current AI-based system have been sourced from a relatively small sample of children (namely, 68 children with ASD and 52 TD children), we believe that they hold promise in capturing language-based markers of ASD from the data, which may be instrumental for their potential usefulness in the early diagnosis of ASD in children. The key potential strength of this model lies in the possibility of stakeholders in the field of ASD (speech and language pathologists, clinicians, special education teachers) to input specific narrative variables from high-risk young children through the use of a tablet-based narrative elicitation application, and then obtain a detailed and objective profile of the child’s language weaknesses. If this method promotes early diagnosis of children with ASD, then such an approach could make a real difference in autism assessment. Our study and the high accuracy of classifiers show that there is potential for the use of a narrative-based ML model in ASD diagnosis. 

The strength of the current study’s narrative-based ML model mainly lies in the fact that the model utilized an AI approach powered by specifically utilizing NLP, employing Open Brain AI’s NLP toolkit [62], BERT semantic embeddings, and ML to provide an analysis of the narrative production and expressive vocabulary of 120 children with and without ASD. Our goal was to determine if specific language measures, identified through NLP, could effectively distinguish between ASD and TD children. 

Children with ASD experience language weaknesses across multiple levels of processing. Specific challenges include difficulties with morphosyntax (grammar), lexical semantics (vocabulary and meaning), and phonology (sound processing) [80,81,82]. They are also impaired in the semantic and pragmatic domain [3,19,26], which has cascading negative effects on the children’s narrative microstructure and macrostructure. Language impairments are a hallmark feature of ASD and are strongly linked to its core traits. Analyzing a child’s language profile has the potential to serve as a red flag, prompting the use of more rigorous diagnostic tools. Unfortunately, this is not yet standard practice, leaving the burden of seeking diagnosis largely on parents and caregivers.

To address this, our approach provided integrated pipelines for eliciting automatic features from text transcripts. These automatic approaches facilitate both micro- and macro-analysis of narratives, and the elicitation of automatic measures that reflect various language aspects of the children’s narrative production, including vocabulary, syntactic structure, and syllabic structure (among others), that may serve to be unique markers of ASD. By enabling early diagnosis, ML models can facilitate timely intervention and support for children with ASD, which could have positive cascading effects on the efficacy of interventions, as well as speech and behavioral therapies.

The advantage of using the automated multimodal approach is that each linguistic analysis provides distinct information about language production in children with ASD. The morphosyntactic features provide information about the narratives’ grammatical structures and how children form sentences while telling a story. The approach provides quantified textual information into the model about the usage of pronouns (e.g., using “I” instead of “s/he”), atypical use of verb tenses or word order, and echolalia (repeating phrases). We also integrated automated information about the lexical characteristics of narration into the model, including impairment in appropriate vocabulary use, unusual or idiosyncratic word usage, and difficulty producing abstract concepts. Our automated approach has also utilized semantic and pragmatic measures mined from a Bidirectional Encoder Representations from Transformers (BERT) AI language model. The BERT embedding is a powerful language model that captures the rich contextual information about words and their relationships within sentences. BERT embedding can identify the minute differences in language usage that might not be apparent in simple word counts or grammatical analysis. Also, embeddings reflect word meanings and how they relate, potentially revealing differences in how children with ASD use language.

### 4.2. Implications of Findings

Overall, the high diagnostic accuracy of the current automated multimodal approach to ASD children’s narrative and vocabulary production could be integrated in several complementary diagnostic tools that are already available for ASD. Ref. [83] presents an extensive examination of psychodiagnostic tools recommended for early ASD screening, categorized into level 1 and level 2 screeners based on their focus and age appropriateness. Level 1 screeners are tailored for younger children, emphasizing social communication, sensory-regulatory functions, and a broad spectrum of behaviors. In contrast, level 2 screeners encompass broader age ranges and delve into specific skills such as communication, social interaction, play, and behavioral patterns. Integrating a narrative evaluation into this diagnostic framework could serve as a complementary tool alongside established instruments like the ADOS [75] or the ADI—R [54]. While the latter tools scrutinize specific behaviors and interactions linked to ASD diagnosis, narrative assessment offers nuanced insights into a child’s language development, social communication, and narrative abilities. This integration could enrich diagnostic assessments by providing a more holistic understanding of early ASD indicators.

### 4.3. Limitations and Future Research

The primary limitation of this study has been the inclusion of children across a wide age range (4–10 years), which could potentially mask the model’s accuracy at specific developmental stages. Studies with larger samples of children in specific age groups (e.g., early childhood, middle childhood) would enable researchers to determine when these language-focused ML models are most accurate. This information would be invaluable for optimizing their use in a clinical setting. Also, it would be important to investigate if any other factors could also affect the classification models, including the children’s socioeconomic status. Also, exploring the model’s performance across different IQ profiles within the ASD population could further refine its applicability for diverse diagnostic contexts. 

Building upon the current work, in our future research, we plan to develop even more precise and age-appropriate AI-powered tools supporting ASD diagnosis. Specifically, we plan to replicate the study with larger sample sizes and ASD children representing a wider range of socioeconomic backgrounds, intellectual abilities, and cultural/linguistic experiences. We will also assess whether the model’s accuracy holds true when targeting other language domains, such as pragmatics (social use of language), receptive language (understanding), as well as language expressions that are related to children’s socio-cognitive and theory of mind skills (e.g., emotion recognition of story characters, production of epistemic verbs like “believe” and “think”) [84]. Finally, the ML models employed here will be tested for their efficacy and efficiency as AI tools within a school setting for early ASD screening, since a further objective is to examine how information from these language assessments can be leveraged by educators and speech therapists to personalize interventions and educational support for children with ASD.

## 5. Conclusions

In this study, we employed ML algorithms to analyze children’s narrative production and expressive vocabulary, aiming to distinguish between ASD and TD children. Our findings highlight the superior performance of two algorithms, namely, hist gradient boosting and XGBoost, which demonstrated high sensitivity and specificity in classifying children with ASD. Given the efficiency, accessibility, and ease of administration of the narrative production and expressive vocabulary assessments (approximately 25 min combined), our automated approach can streamline the diagnostic process and expedite the initiation of targeted interventions. As such, our model may represent a key step towards expediting the diagnosis of ASD as well as accelerating the delivery of interventions at earlier and more impactful stages of child development.

## Figures and Tables

**Table 1:** ASD and TD children’s demographic characteristics (means, standard deviations in parentheses, and ranges).

Group	Age	FSIQ
ASD	8;7 (*4.6*) 4;2–10;7	84.1 (*18.1*) 71–120
TD	8;4 (*4.3*) 4;2–10;6	82.7 (*17.6*) 72–121

Notes: ASD = autism spectrum disorder; TD = typically-developing; FSIQ = Full-Scale IQ.

**Table 2:** Characteristics of each of the ML models employed.

Model	Type	Characteristics
Gradient Boosting	Ensemble	Sequential, combines multiple weak learners
Decision Trees	Tree-like structure	Interpretable, key features important
Hist Gradient Boosting	Variant of gradient boosting	Uses histograms to represent features, efficient
XGBoost	Implementation of gradient boosting	Speed, accuracy, techniques to improve performance

**Table 3:** Accuracy, F1 and ROC/AUC outcomes scores.

Model	Accuracy	F1	ROC AUC
Gradient Boosting	0.925	0.926829	0.9425
Decision Trees	0.925	0.926829	0.9250
Hist Gradient Boosting	0.975	0.974359	0.9675
XGBoost	0.975	0.974359	0.9600

## Data Availability

Our NLP analysis system is available at Open Brain AI (https://openbrainai.com) (10 December 2023). Data are available only after request from C.K.T.
